# Angiotensin II differentially affects hippocampal glial inflammatory markers in young adult male and female mice

**DOI:** 10.1101/lm.053507.121

**Published:** 2022-09

**Authors:** Teresa A. Milner, Ryan X. Chen, Diedreanna Welington, Batsheva R. Rubin, Natalina H. Contoreggi, Megan A. Johnson, Sanoara Mazid, Jose Marques-Lopes, Roberta Marongiu, Michael J. Glass

**Affiliations:** 1 Feil Family Brain and Mind Research Institute, Weill Cornell Medicine, New York, New York 10065, USA; 2 Harold and Milliken Hatch Laboratory of Neuroendocrinology, The Rockefeller University, New York, New York 10065, USA; 3 Neurological Surgery Department, Weill Cornell Medicine, New York, New York 10065, USA

## Abstract

Hypertension is a risk factor for neurodegenerative disorders involving inflammation and inflammatory cytokine-producing brain cells (microglia and astrocytes) in the hippocampus and medial prefrontal cortex (mPFC). Here we investigated the effect of slow-pressor angiotensin II (AngII) on gliosis in the hippocampus and mPFC of young adult (2-mo-old) male and female mice. In males, AngII induced hypertension, and this resulted in an increase in the density of the astrocyte marker glial fibrillary acidic protein (GFAP) in the subgranular hilus and a decrease in the density of the microglial marker ionized calcium binding adapter molecule (Iba-1) in the CA1 region. Females infused with AngII did not show hypertension but, significantly, showed alterations in hippocampal glial activation. Compared with vehicle, AngII-infused female mice had an increased density of Iba-1 in the dentate gyrus and CA2/3a region. Like males, females infused with AngII exhibited decreased Iba-1 in the CA1 region. Neither male nor female mice showed differences in GFAP or Iba-1 in the mPFC following AngII infusion. These results demonstrate that the hippocampus is particularly vulnerable to AngII in young adulthood. Differences in gonadal hormones or the sensitivity to AngII hypertension may account for divergences in GFAP and Iba-1 in males and females.

Hypertension is a significant risk factor for neurological disorders such as Alzheimer's disease (AD) that are associated with neurodegeneration and cognitive decline (Daugherty 2021). Hypertension can develop during the life span yet is often studied at middle and late life. There is emerging evidence that hypertension is becoming more common in late adolescence and early adulthood (Azegami et al. 2021; Hamrahian and Falkner 2022). In addition, there is increasing awareness that the duration of hypertension can impact the onset of neural degeneration (Schaare et al. 2019; Yang et al. 2021) and cognitive dysfunction (Yaffe et al. 2014, 2021; Mahinrad et al. 2020; Zhou et al. 2022). Although the age of onset of hypertension may influence the trajectory of degenerative disease in later life, the effect of hypertension on brain health in young adult subjects is relatively underinvestigated.

Hippocampal and medial prefrontal cortical pathology are commonly present in neurodegenerative diseases like AD (Belonwu et al. 2021). Structurally and functionally, both the hippocampus and mPFC also are compromised during hypertension (Raz et al. 2007; Gonzalez et al. 2015; Bu et al. 2018). In the hippocampus, hypertension is known to disrupt cerebrovascular function, promote inflammatory processes, and contribute to neuronal impairment and cognitive decline (Iulita et al. 2018). Although less studied than the hippocampus, the PFC is also compromised by hypertension (Raz et al. 2007; Bu et al. 2018; Wang et al. 2020).

Microglia, the resident macrophages in the brain, have been implicated in inflammatory states, cognitive function (Cornell et al. 2022), and the brain's response to hypertension (Calvillo et al. 2019; Li et al. 2020). An increase in the density of ionized calcium binding adapter molecule (Iba-1), a protein constitutively expressed in microglia and up-regulated when microglia enter an activated stage (Imai et al. 1996; Sasaki et al. 2001), is commonly reported in models of cognitive and neurodegenerative disorders (Prinz et al. 2021).

In addition to microglia, astrocytes also have been implicated in the emergence of hippocampal and cortical dysfunction. Astrocytes play critical roles in blood–brain barrier (BBB) formation; brain metabolic, ion, and water homeostasis; neurotransmitter recycling; synapse formation; and neuroimmune signaling (Matias et al. 2019). In the context of insult, pathogen infection, or neurological disease, astrocytes undergo functionally complex reactive responses (Chiu et al. 2014; Giovannoni and Quintana 2020) that are associated with an increase in glial fibrillary acidic protein (GFAP) gene and protein expression (Crespo-Castrillo et al. 2020; Sofroniew 2020).

To better understand the consequences of elevated blood pressure on the young adult brain, we conducted an exploratory investigation of the impact of hypertension on the expression of microglia and astrocyte markers—Iba-1 and GFAP, respectively—in the hippocampus and mPFC of male mice. Mice were exposed to angiotensin II (AngII) using the “slow-pressor” model (Dickinson and Lawrence 1963), which in males mimics the gradual rise in blood pressure and increase in sympathetic activation (Grassi and Ram 2016; Lerman et al. 2019) characteristic of essential hypertension (Lerman et al. 2019). Significantly, there is an important sex dimorphism in the risk for hypertension. Compared with men, women are protected from hypertension before middle age but become increasingly affected as they reach perimenopause, and intact young female rodents show a reduced sensitivity to AngII hypertension (Van Kempen et al. 2016). Similarly, there are sex differences in the incidence, progression, and severity of hypertension-associated neurodegenerative disease (Lopez-Lee et al. 2021). Furthermore, sex differences in glial function have also been documented within the context of neurodegenerative diseases (Kodama and Gan 2019; Biechele et al. 2020). Given this evidence, the effect of AngII on hippocampal and medial prefrontal cortical glial markers also was investigated in young intact female mice.

## Results

### AngII increases systolic blood pressure (SBP) in males but not females

**Figure 1.**
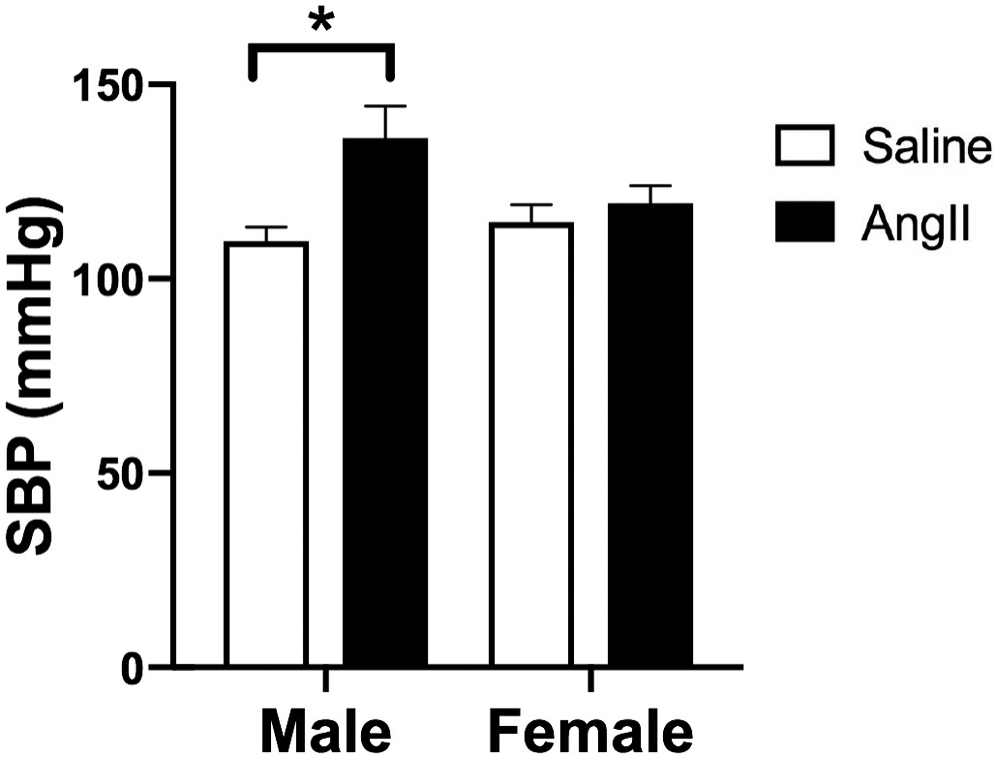
Systolic blood pressure in young male and female mice following saline or AngII. SBP was increased in Ang males ([*] *P* < 0.05), but not Ang females, relative to Sal-infused controls on day 13 of infusion. *N* = 6 mice per group.

There were no significant differences in SBP in males infused with saline or AngII (Sal males and Ang males, respectively) or in females treated with saline or AngII (Sal females and Ang females, respectively) prior to implanting osmotic minipumps (i.e., baseline measurements) (data not shown). However, SBP was significantly increased in Ang males, but not Ang females, relative to Sal-infused controls on day 13 of treatment [*F*_(3,20)_ = 4.36, *P* = 0.016] (Fig. 1).

### Iba1: hippocampus

**Figure 2.**
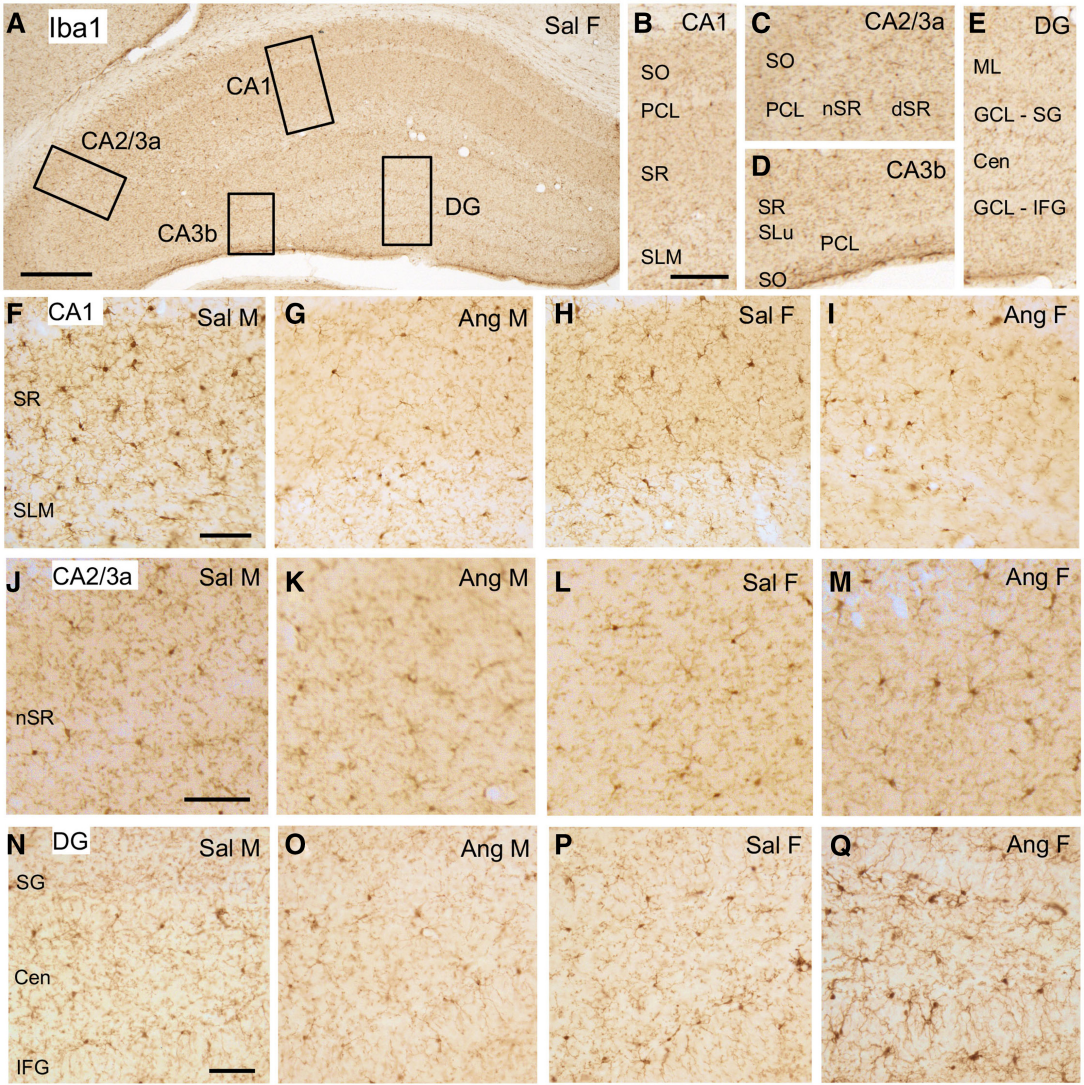
Iba1 labeling in the dorsal hippocampus. (*A*) Low-magnification photomicrograph of Iba1 labeling the dorsal hippocampus. Boxes indicate regions of the CA1, CA2/3a, CA3b, and dentate gyrus (DG) that were sampled. (*B*) Enlargement of the boxed region shown in CA1. (PCL) Pyramidal cell layer, (SLM) stratum lacunosum–moleculare, (SO) stratum oriens, (SR) stratum radiatum. (*C*) Enlargement of the boxed region shown in CA2/3a. (dSR) Distal SR, (nSR) near stratum radiatum. (*D*) Enlargement of the boxed region in CA3b. (SLu) Stratum lucidum. (*E*) Enlargement of the boxed region shown in the DG. (Cen) Central hilus, (GCL) granule cell layer, (IFG) infragranular layer, (ml) molecular layer, (SG) supragranular layer. (*F*–*I*) Representative micrographs showing Iba1 labeling in the CA1 of a Sal male (*F*), Ang male (*G*), Sal female (*H*), and Ang female (*I*). (*J*–*M*) Representative micrographs showing Iba1 labeling in the CA1 of a Sal male (*J*), Ang male (*K*), Sal female (*L*), and Ang female (*M*). (*N*–*Q*) Representative micrographs showing Iba1 labeling in the DG of a Sal male (*N*), Ang male (*O*), Sal female (*P*), and Ang female (*Q*). Scale bars: *A*, 250 µm; *B*–*E*, 100 µm; *F*–*Q*, 50 µm.

As illustrated in an example from a Sal female, Iba1-labeled cells were found scattered throughout all lamina in the CA1, CA3, and dentate gyrus (DG); however, fewer Iba1-labeled cells were found in the pyramidal and granule cell layers (Fig. 2A–E). Representative micrographs showing the distribution of Iba1 labeling in the CA1, CA2/3a, and DG regions from each of the four animal groups are shown in Figure 2, F–Q (CA3b not shown).

**Figure 3.**
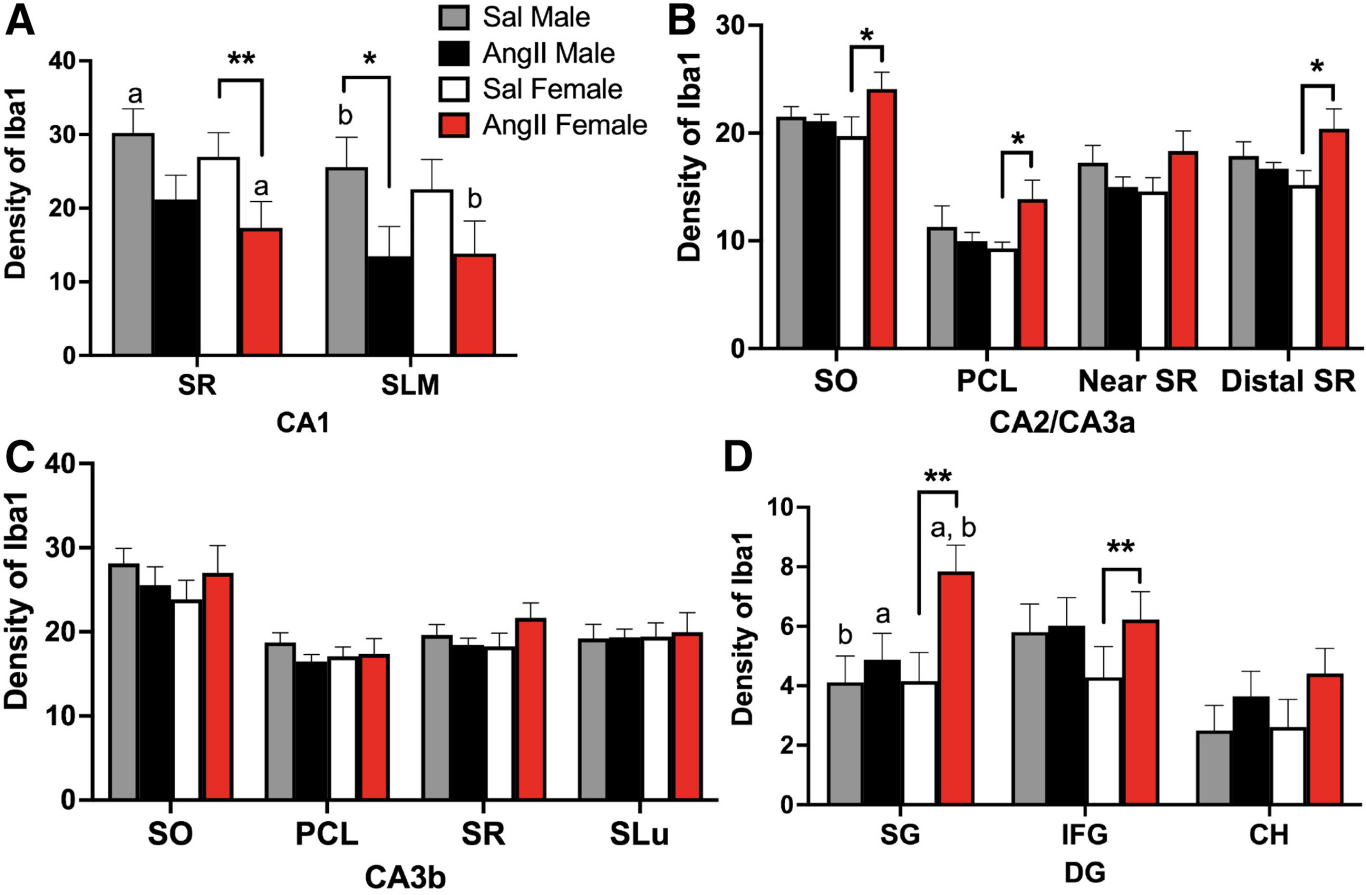
Sex differences in the Iba1 labeling in the dorsal hippocampus following AngII. (*A*) The density of Iba1 labeling in CA1 in the SR and SLM is decreased in Ang females compared with Sal females and Sal males. (^*,a,b^) *P* < 0.05; (**) *P* = 0.005. (*B*) In CA2/CA3a, the densities of Iba1 labeling in the SO, PCL, near SR, and distal SR are elevated in Ang females compared with Sal females. Ang females also have elevated densities of Iba1 labeling in the PCL and distal SR compared with Ang males. (^*,a,b^) *P* < 0.05. (*C*) In CA3b, there are no significant differences in Iba1 densities between the four groups in any lamina. (*D*) In the DG, Ang females compared with Sal females have greater densities of Iba1 labeling in the SG and IFG regions. (^a,b^) *P* < 0.05; (**) *P* = 0.01). *N* = 6 mice per group.

The density of Iba1 labeling in all subregions of the CA1 and DG analyzed was not significantly different between Sal males and Sal females (Fig. 3A,D). However, sex differences in the density of Iba1 labeling emerged in all subregions except CA3b following AngII infusion. In the CA1 region, Ang males had a lower density of Iba1-ir in the stratum lacunosum–moleculare (SLM) compared with Sal males [*t*_(29)_ = 2.08, *P* = 0.0427] (Fig. 3A). Moreover, Ang females had less dense Iba1-ir in stratum radiatum (SR) compared with both Sal females [*t*_(29)_ = 2.96, *P* = 0.0056] (Fig. 3A) and Sal males [*t*_(29)_ = 2.71, *P* = 0.0127] (Fig. 3A). There were no significant differences in the number of Iba1-labeled cell bodies between Sal males and Ang males or Sal females and Ang females in either the SR or SLM (Table 1).

**Table 1.**
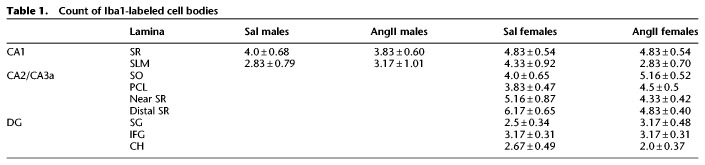
Count of Iba1-labeled cell bodies

Significant differences in the density of Iba-1 labeling following AngII infusion were seen in the CA2 and CA3 areas only in female mice. In the CA2/CA3a region, the density of Iba-1 was higher in the stratum oriens [SO; *t*_(19)_ = 2.30, *P* = 0.0327], PCL [*t*_(19)_ = 2.30, *P* = 0.0324], and distal SR [*t*_(19)_ = 2.76, *P* = 0.0124] in Ang females compared with Sal females (Fig. 3B). Additionally, the density of Iba1 in the pyramidal cell layer [PCL; *t*_(19)_ = 1.96, *P* = 0.0642] and distal SR [*t*_(19)_ = 1.97, *P* = 0.0641] tended to be higher in Ang females compared with Ang males. There were no significant differences in the density of Iba1 in any of the subregions of CA3b in either males or females following AngII infusion (Fig. 3C). In areas showing increased Iba1 density, counts of the number of cells labeled for Iba also were made. There were no significant differences in the number of Iba1-labeled cell bodies between Sal females and Ang females in any lamina in CA2/3a (Table 1).

In the DG, there were no significant differences in the density of Iba1 in either the supragranular (SG) or infragranular (IFG) regions of Sal males compared with Ang males. However, the density of Iba1-ir was significantly higher in the SG region in Ang females compared with Sal females [*t*_(29)_ = 2.79, *P* = 0.0091] (Fig. 3D) as well as Sal males [*t*_(29)_ = 2.96, *P* = 0.006] (Fig. 3D) and Ang males [*t*_(29)_ = 2.54, *P* = 0.0254] (Fig. 3D). Similarly, the density of Iba1-ir was higher in the IFG of Ang females compared with Sal females [*t*_(29)_ = 2.07, *P* = 0.0474] (Fig. 3D). There were no significant differences in the number of Iba1-labeled cell bodies between Sal females and Ang females in any lamina in the DG (Table 1).

### Iba1: mPFC

**Figure 4.**
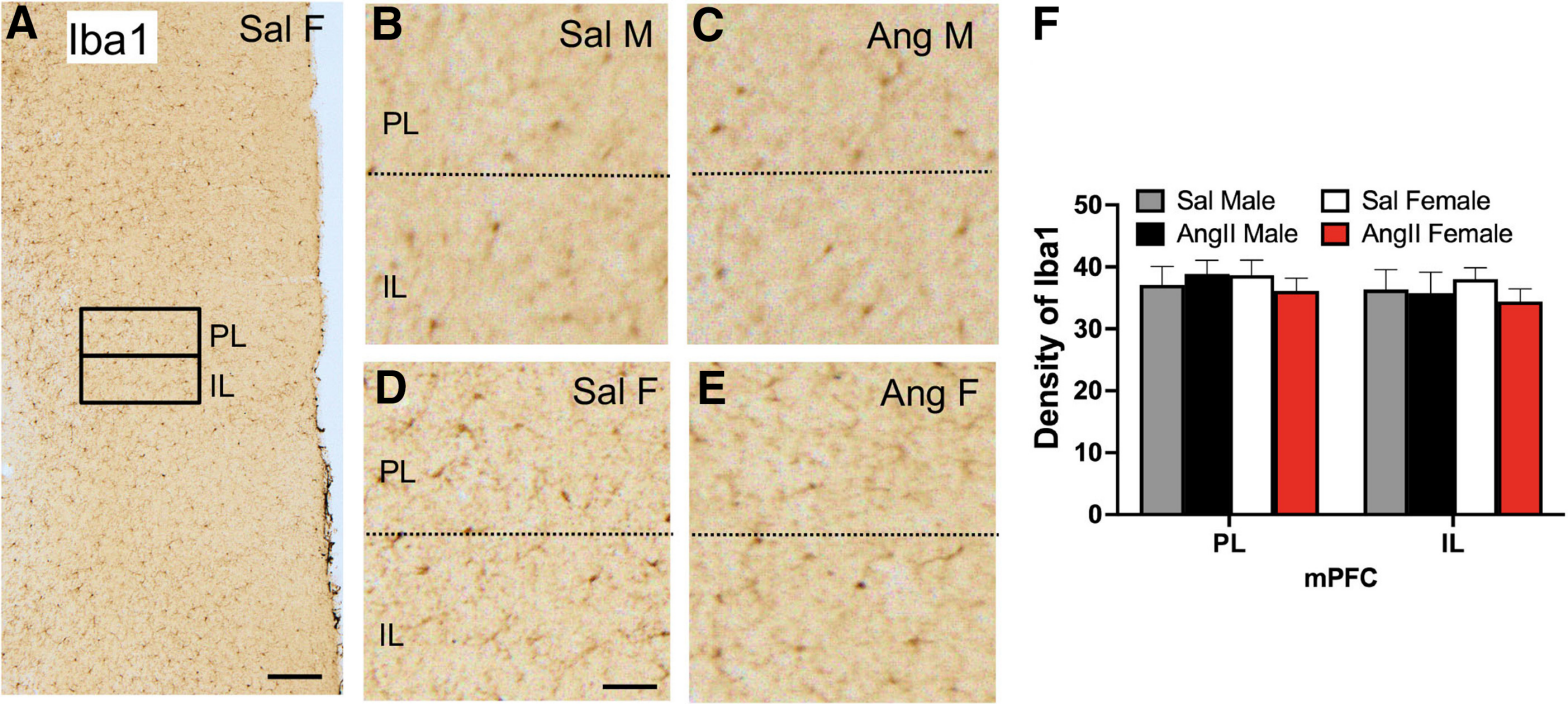
The expression of Iba1 in the mPFC is not altered by either sex or slow-pressor AngII. (*A*) Low-magnification photomicrograph of Iba1 labeling the mPFC shows regions of the PL and IL selected for densitometry (example from Sal female). (*B*–*E*) Representative micrographs showing Iba1 labeling in the PL and IL of a Sal male (*B*), Ang male (*C*), Sal female (*D*), and Ang female (*E*). Scale bars: *A*, 250 µm; *B*–F, 50 µm. (*F*) There are no significant differences in the density of Iba1 labeling in the PL and IL layers of the mPFC between any group. *N* = 6 mice per group.

The density of Iba1 labeling was sampled from the prelimbic (PL) and infralimbic (IL) regions of the mPFC (Fig. 4A). As shown in examples from all four experimental groups (Fig. 4B–E), Iba1-labeled cells were found sparsely scattered throughout the PL and IL regions. Unlike the hippocampus, there were no significant differences in the density of Iba1 labeling in either the PL or IL in any group (Fig. 4F).

### GFAP: hippocampus

**Figure 5.**
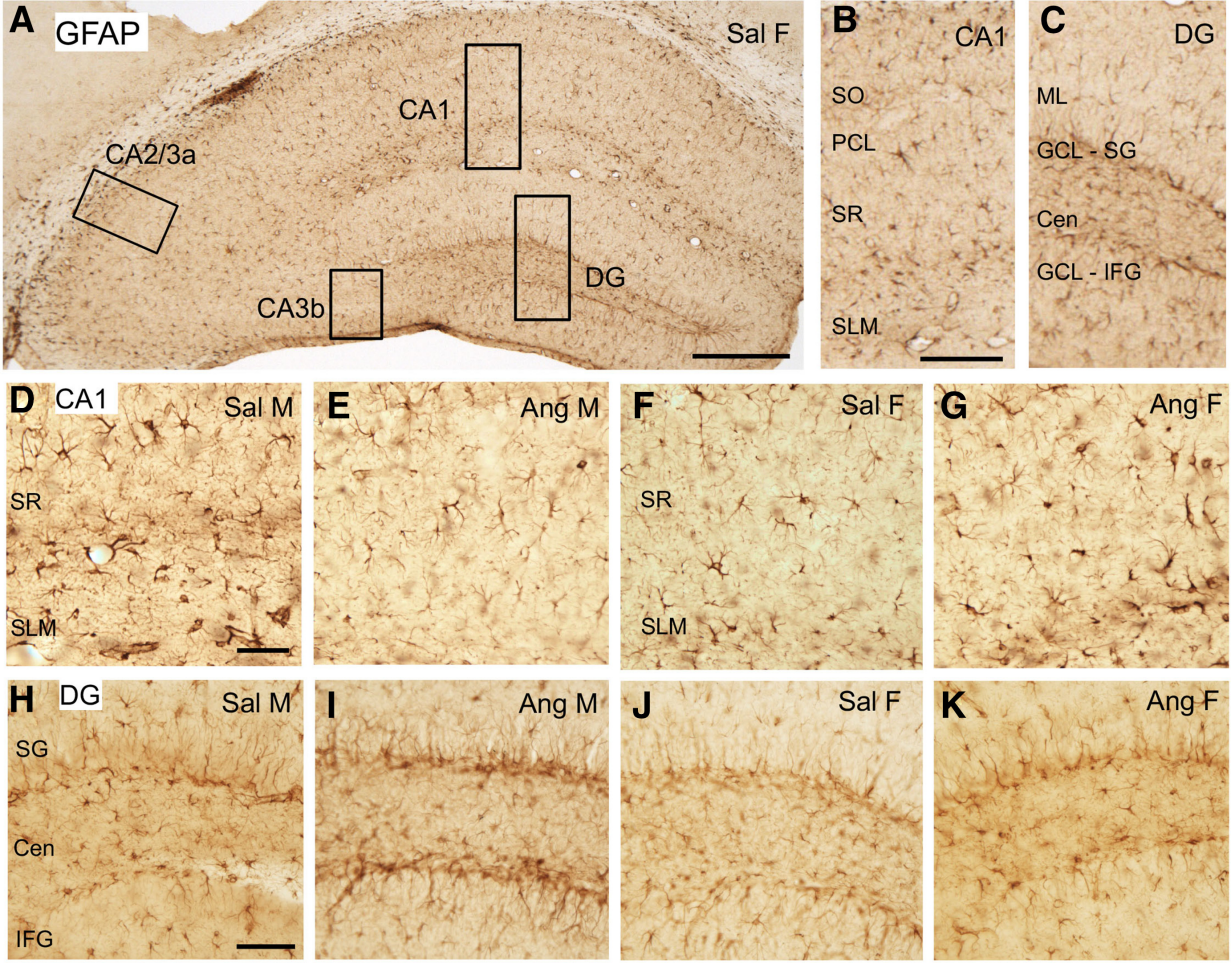
GFAP localization in the dorsal hippocampus. (*A*) Low-magnification photomicrograph of GFAP labeling the dorsal hippocampus. Boxes indicate regions of the CA1, CA2/3a, CA3b, and DG that were sampled. (*B*) Enlargement of the boxed region shown in CA1. (PCL) Pyramidal cell layer, (SLM) stratum lacunosum–moleculare, (SO) stratum oriens, (SR) stratum radiatum. (*C*) Enlargement of the boxed region shown in the DG. (Cen) Central hilus, (IFG) infragranular layer, (SG) supragranular layer. (*D*–*G*) Representative micrographs showing GFAP labeling in the CA1 of a Sal male (*D*), Ang male (*E*), Sal female (*F*), and Ang female (*G*). (*H*–*K*) Representative micrographs showing GFAP labeling in the DG of a Sal male (*H*), Ang male (*I*), Sal female (*J*), and Ang female (*K*). Scale bars: *A*, 250 µm; *B*,*C*, 100 µm; *D*–*K*, 50 µm.

GFAP-labeled cells were found throughout the CA1, CA3, and DG but were particularly dense in the CA1 SLM region as well as the hilus of the DG (Fig. 5A–C). Representative micrographs showing the distribution of GFAP labeling in the CA1 and DG regions from the four groups are shown in Figure 5, D–K.

**Figure 6.**
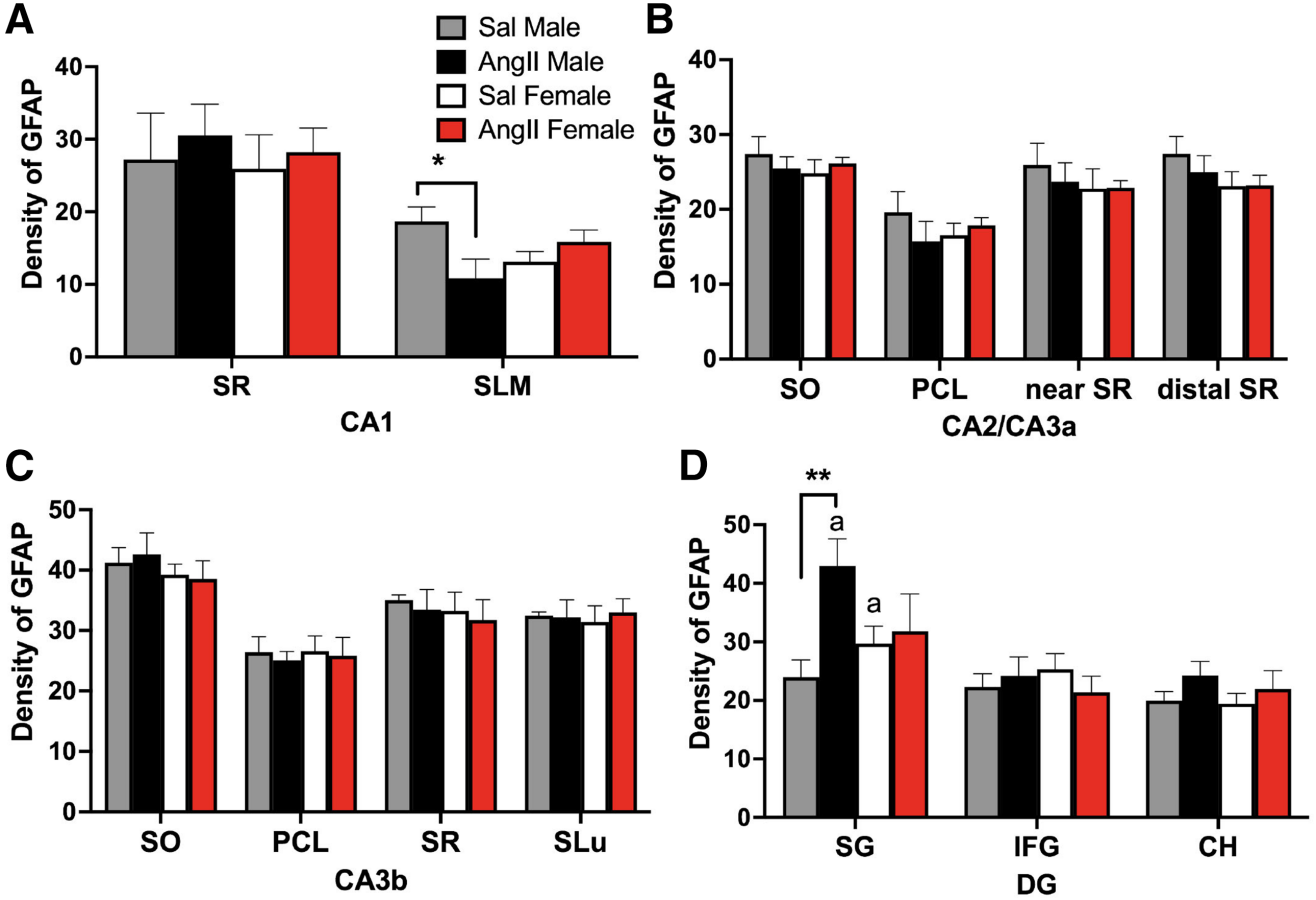
Sex differences in the GFAP labeling in the hippocampus following slow-pressor AngII. (*A*) The density of GFAP labeling in CA1 in the SLM is decreased in Ang males compared with Sal males. (*) *P* < 0.05. (*B*,*C*) In CA2/CA3a and CA3b, the densities of GFAP labeling were not different between the four groups in any lamina. (*D*) In the DG, Ang males have greater densities of GFAP labeling in the SG regions compared with Sal males and Sal females. (**) *P* = 0.01. *N* = 6 mice per group.

The density of GFAP labeling in all subregions of the CA1, CA2/3a, CA3b, and DG analyzed was not significantly different between Sal males and Sal females (Fig. 6). However, a few sex differences in the density of GFAP labeling were observed in both regions following AngII infusion. In CA1, the density of GFAP-ir was significantly lower in Ang males compared with Sal males [*t*_(29)_ = 2.317, *P* = 0.0277] (Fig. 6A). In the DG, the density of GFAP-ir was higher in the SG in Ang males compared with Sal males [*t*_(29)_ = 3.178, *P* = 0.0035] (Fig. 6D) and Sal females [*t*_(29)_ = 1.963, *P* = 0.0592] (Fig. 6D). In Ang males, elevated GFAP labeling was particularly prominent in the subgranular hilus of the SG (Fig. 5I). There were no significant differences in the density of GFAP in any of the subregions of CA2/3a and CA3b in either females or males administered Sal or AngII (Fig. 6B,C).

### GFAP: mPFC

**Figure 7.**
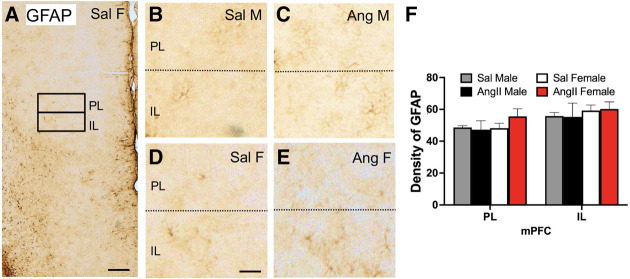
The expression of GFAP in the mPFC is not altered by either sex or AngII. (*A*) Low-magnification photomicrograph of GFAP labeling the mPFC shows regions of the PL and IL selected for densitometry (example from Sal female). (*B*–*E*) Representative micrographs showing GFAP labeling in the PL and IL of a Sal male (*B*), Ang male (*C*), Sal female (*D*), and Ang female (*E*). Scale bars: *A*, 250 µm; *B*–*F*, 50 µm. (*F*) There are no significant differences in the density of GFAP labeling in the PL and IL layers of the mPFC between any group. *N* = 6 mice per group.

The density of GFAP labeling was sampled from the PL and IL regions of the mPFC (Fig. 7A). As shown in examples from all four experimental groups (Fig. 7B–E), few GFAP-labeled cells were found in the PL and IL regions. There were no significant differences in the density of GFAP labeling in either the PL or IL in any group (Fig. 7F).

## Discussion

We report that hypertension induced by slow-pressor infusion of AngII in male mice results in altered expression of markers of glial reactivity in select hippocampal subregions. Male mice treated with AngII showed an increase in the density of the astrocyte marker GFAP that was restricted to the subgranular hilus, and a small but significant decrease in GFAP in the SLM. In contrast, female mice infused with AngII did not exhibit hypertension, but did show an increase in the density of Iba1 immunoreactivity, which was limited to select areas of the CA2/CA3a and DG. Neither males nor females differed in the expression of glia markers in the mPFC following AngII. These results demonstrate that male mice express hypertension following AngII, and an increase in glial activation marked by expression of GFAP in select hippocampal subregions. In contrast, female mice exhibit signs of microglia activation in response to AngII even in the absence of increased blood pressure.

Because the hippocampus is a structurally and functionally complex brain area, we quantified the density of glial marker immunoreactivity in different subregions, as astrocytes and microglia are known to have region-specific susceptibility to different insults (Lana et al. 2021), particularly in the context of neurodegenerative diseases associated with cognitive impairment (Su et al. 2018; Dounavi et al. 2020; McKeever et al. 2020). Similarly, the PL and IL regions of the mPFC were selected for analysis because both subregions are involved in cognition; the PL is associated with behavioral flexibility, whereas the IL is implicated in impulsive behavior as well as habit formation (van Aerde et al. 2008).

In males, the altered density of hippocampal GFAP was not uniformly distributed throughout the hippocampus but was restricted to select hippocampal subregions. Specifically, there was an increase in GFAP in the subgranular hilus, in which cells undergoing adult neurogenesis are found (Araki et al. 2020). Moreover, AngII-treated males had a small but significant decrease in GFAP in the SLM, which receives entorhinal and thalamic afferents and contains interneurons important for rhythmic synchronization of pyramidal cells thought to contribute to mnemonic processes (Chapman and Lacaille 1999; Vu et al. 2020). In addition to GFAP, AngII-infused male mice also showed decreases in the density of Iba-1 labeling in the CA1 SLM.

In contrast to males, it was found that AngII infusion did not elicit an increase in blood pressure in female mice, consistent with other reports (Xue et al. 2005; Girouard et al. 2009; Marques-Lopes et al. 2017; Milner et al. 2021). Like male mice, female mice did not show differences in either the density of Iba1 or GFAP labeling in the mPFC. However, unlike male mice, female mice did not show changes in GFAP in any region of the hippocampus. In contrast, female AngII-treated mice exhibited alterations in the density of Iba-1 labeling in the hippocampus. Robust increases in the microglial marker were seen in all lamina of the CA2/CA3a, regions that are innervated by the hypothalamic supramammillary nucleus, believed to participate in theta rhythms, a process important for encoding new memories (Jones and McHugh 2011). Moreover, Iba1 was elevated in AngII-infused females in the infragranular and subgranular zones of the DG—areas in which cells undergoing adult neurogenesis are concentrated (Araki et al. 2020).

The basis for the differing patterns of glial activation in the hippocampus of male and female mice is uncertain but may be related to the actions of gonadal hormones (Conejo et al. 2005; Arias et al. 2009). For example, changes in the density of GFAP following AngII infusion in male mice may be related to the actions of androgens. Testosterone has been shown to regulate GFAP levels in the hippocampus of males during postnatal development (Conejo et al. 2005) and into adulthood (McQueen et al. 1992). Serum testosterone also has been shown to be inversely correlated with hippocampal GFAP mRNA in males (Nichols et al. 1993). Microglia also are impacted by gonadal hormones in females. For example, ovariectomy is associated with an increase in Iba1 in middle-aged mice (Sárvári et al. 2017), and signs of microglia reactivity are decreased by estradiol in the hippocampus of aged ovariectomized mice (Lei et al. 2003). In addition, ovariectomy has been reported to result in an increase in labeling of macrophage antigen complex-1, a marker of reactive microglia, in the hippocampus of aged mice (Benedusi et al. 2012). Additionally, gonadal hormones impact microglia in the context of neurodegenerative disease in females, as indicated by findings that chronic estrogen deficiency is associated with increased microglial activation and neurodegeneration in a mouse AD model (Prat et al. 2011).

Differences in the density of hippocampal glial markers in males and females may also be due to differences in blood pressure following AngII. In males, there is evidence that AngII, is capable of elevating blood pressure by acting on circumventricular organs (CVOs), which in turn have direct neural projections to hypothalamic circuits (Mangiapane and Simpson 1980; Lind et al. 1983; Ferguson 2009). One well-characterized CVO–hypothalamic pathway implicated in AngII-mediated hypertension involves an excitatory pathway between the subfornical organ and the paraventricular nucleus (PVN) of the hypothalamus, a critical regulator of sympathetic output involving brainstem and spinal cord circuits that play an important role in blood pressure regulation (Mangiapane and Simpson 1980; Lind et al. 1983; Ferguson 2009). The increase in blood pressure may in turn affect cerebral blood flow, oxygenation, and neurovascular coupling, and lead to oxidative stress and neuroinflammation (Iadecola and Gottesman 2019). Alternatively, young gonadally intact female mice are protected from AngII hypertension (Xue et al. 2005; Capone et al. 2009; Marques-Lopes et al. 2017). However, following ovariectomy or accelerated ovarian failure (AOF), animals show an increase in blood pressure following AngII, demonstrating the importance of ovarian hormones in hypertension (Xue et al. 2005; Milner et al. 2021).

Hippocampal microgliosis in the absence of AngII hypertension in intact female mice may be the result of AngII-mediated actions on the cerebral vasculature. For example, it has been reported that following chronic AngII infusion, the BBB in the somatosensory cortex becomes disrupted even when hypertension is prevented (Santisteban et al. 2020). Alternatively, AngII can also interfere with the coordination of neural activity with cerebral blood flow (i.e., neurovascular coupling) even in the absence of hypertension (Capone et al. 2011). Dysregulation of either the BBB or neurovascular coupling in response to AngII may contribute to neuroinflammatory processes in the hippocampus of females even in the absence of hypertension (Schaeffer and Iadecola 2021; Takata et al. 2021).

The impact of altered Iba1 and GFAP on hippocampal function following AngII can only be speculated at present. Both microglia and astrocytes have been well characterized for their roles in neuroinflammatory processes that contribute to neuropathology in the context of brain ischemia, pathogen infection, trauma, and other deleterious states (Li and Barres 2018; Matias et al. 2019; Lana et al. 2021). Therefore, under the influence of persistent inflammatory conditions known to occur during AngII exposure (Tota et al. 2013; Park et al. 2020), glial dysregulation may contribute to neuropathological processes (Arevalo et al. 2013; Spangenberg and Green 2017). Alternatively, glia can exert homeostatic and even protective effects by maintaining metabolic homeostasis, releasing trophic factors, regulating synaptic architecture, or contributing to neurogenesis (Chen and Trapp 2016; Boghdadi et al. 2020). In this context, the increased expression of glial markers may reflect processes that help to shield affected areas of the hippocampus from an emerging toxic environment associated with AngII exposure. Irrespective of the precise functional role of altered glial reactivity, the present finding that slow-pressor AngII infusion is associated with increased Iba1 and GFAP suggests that even relatively low AngII exposure can elicit neural-protective or pathological processes involving glial pathways.

In conclusion, young adult male and female mice show differential alterations in glial activation in the hippocampus, but not mPFC, in response to AngII. A more precise understanding of the relationships between neural health, cognitive function, and glial activity in response to AngII across the sexes awaits further inquiry.

## Materials and Methods

### Animals

This study used young adult (∼2 mo old at the initiation of the experiments) (Flurkey and Currer 2004) male and female C57BL/6 mice (*N* = 24) bred and maintained in a colony at Weill Cornell Medicine (WCM). Mice weighed 23–28 g at the end of the study and were housed in groups of three to four animals per cage and maintained on a 12-h light–dark cycle (lights out 18:00 h) with ad libitum access to water and rodent chow in their home cages. Four groups of mice (*N* = 6/group) were used: (1) saline male, (2) AngII male, (3) saline female, and (4) AngII female. Tissues from these mice were obtained from different cohorts of mice used in our previous studies (Marques-Lopes et al. 2014, 2015; Ovalles et al. 2019). All experiments were approved by the Institutional Animal Care and Use Committees at Weill Cornell Medicine in accordance with guidelines established by the National Institutes of Health Guide for the Care and Use of Laboratory Animals. All efforts were made to minimize the number of animals used and their suffering.

### Estrous cycle determination

Prior to implanting osmotic minipumps (below), daily vaginal smears were taken in female mice for 2 wk between 9:00 a.m. and 10:00 a.m. to determine estrous cycle stage (Turner and Bagnara 1971). To control for the effects of handling, males were removed from their home cage and handled daily. Estrous cycles were 4–5 d long and consisted of three primary phases: proestrus (high estrogen levels; 0.5–1 d), estrus (declining estrogen levels; 2–2.5 d), and diestrus (low estrogen and progesterone levels; 2–2.5 d). Only mice with at least two regular estrous cycles were used in the study (Marques-Lopes et al. 2014, 2015). Additionally, a terminal vaginal smear was taken on the day of euthanasia to assess final estrous cycle phase as determined by cytological examination. The young female mice used in this study were in estrus or diestrus on the day of euthanasia (Marques-Lopes et al. 2015; Ovalles et al. 2019).

### AngII infusion and blood pressure measurement

Mice were handled by the same investigator or investigators for each experimental procedure. Seven days to 10 d prior to implanting osmotic minipumps, mice were habituated to the blood pressure measurement conditions, including handling and exposure to the apparatus. As described in prior studies (Woods et al. 2020; Milner et al. 2021), mice were anesthetized with isoflurane and implanted subcutaneously in the upper back with osmotic minipumps (Alzet) containing a saline vehicle (saline with 0.1% bovine serum albumin [BSA] added to prevent peptide adherence to the pumping chamber) or AngII dissolved in this saline vehicle (600 ng/kg^−1^/min^−1^) for delivery over 14 d. Systolic blood pressure (SBP) was measured during the light period (3:00 p.m.–4:00 p.m.) before (baseline) and 2, 5, 9, and 13 d after minipump implantation in awake mice using a Hatteras MC-4000 tail-cuff blood pressure system. In each blood pressure assessment session, a total of 10–20 blood pressure measurements was recorded over a 10-min period. Blood pressure measurements were averaged for each mouse, which were then combined to generate group mean SBP values across treatments. (Note that as the tissues used in this study were obtained from several cohorts of mice used in prior studies, a new group mean SBP was generated.)

### Antibodies

A rabbit polyclonal antibody to GFAP (Abcam ab7260, lot GR20948-21, RRID: AB_305808) raised against a full-length protein corresponding to human GFAP was used. On Western blot, this antibody recognized a band of 55 kDa and a 48-kDa band corresponding to GFAP (manufacturer's datasheet). A rabbit polyclonal antibody raised to a synthetic peptide corresponding to the C terminus of Iba1 (Fujifilm Wako Pure Chemical Corporation SAR6502, 019-19741) was used. The antibody is reactive with human, mouse, and rat iba1 and recognizes a 17-kDa band protein on Western blot (manufacturer's datasheet).

### Light microscopic immunocytochemistry

Mice were processed for immunocytochemistry using established procedures (Milner et al. 2011). Mice were deeply anesthetized with 150 mg/kg sodium pentobarbital i.p., and their brains were fixed by aortic arch perfusion sequentially with 2–3 mL of normal saline (0.9%) containing 2% heparin followed by 30 mL of 3.75% acrolein (Polysciences; now discontinued) and 2% paraformaldehyde in 0.1 M phosphate buffer (PB; pH 7.4). After dissection from the cranium, each brain was postfixed in 1.9% acrolein and 2% paraformaldehyde in PB for 30 min. The forebrain containing the hippocampus was sectioned (40 µm thick) using a vibratome (Leica Microsystems VT1000X). Brain sections were stored at −20°C in cryoprotectant (30% sucrose, 30% ethylene glycol in PB) until immunocytochemical processing.

For each brain region (hippocampus or mPFC), a single cohort of sections from mice from each treatment group was processed for Iba1 or GFAP (*N* = 6 per experimental condition). For this, one dorsal hippocampal or medial prefrontal cortical section from each animal was matched with regards to rostrocaudal level (hippocampus: −2.00 to −2.70 mm from bregma; mPFC: +1.4 to +1.7 from bregma) (Hof et al. 2000) and then punch-coded in the cortex. Tissue sections from each treatment condition then were pooled into single containers to ensure that sections from each experimental cohort were identically exposed to reagents (Milner et al. 2011). Next, sections were treated with 1% sodium borohydride in PB for 30 min to neutralize reactive aldehydes and rinsed eight to 10 times in PB until gaseous bubbles disappeared. Sections then were transferred to 0.1 M Tris saline (TS; pH 7.6) followed by an incubation in 0.5% BSA in TS for 30 min to minimize nonspecific labeling. Sections were incubated in primary Iba-1 (1:4000 dilution) or GFAP (1:6000 dilution) antisera diluted in 0.1% Triton-X and 0.1% BSA in TS for 1 d at room temperature and 1 d at −4°C. Sections then were washed in TS and incubated in goat antirabbit IgG conjugated to biotin (Jackson ImmunoResearch, Inc., 111-065-144, RRID: AB_2337965) in 0.1% BSA and TS. Next, sections were rinsed in TS and incubated with avidin biotin complex (ABC) diluted to half of the manufacturer's recommended dilution (Vectastain Elite kit, Vector Laboratories) for 30 min. After rinsing in TS, the bound peroxidase was visualized by reaction in 3,3′-diaminobenzidine (Sigma-Aldrich) and 0.003% hydrogen peroxide in TS for 3 min (GFAP hippocampus), 4 min (GFAP mPFC), or 7 min (iba1, hippocampus, and mPFC). All primary and secondary antibody incubations were carried out at 145 rpm, whereas all rinses were conducted at 90 rpm on a rotator shaker. Sections were mounted from 0.05 M PB onto gelatin-coated glass slides, dehydrated through an ascending series of alcohol through xylene, and coverslipped with DPX (Sigma-Aldrich).

### Analysis and figure preparation

The analysis was performed by investigators blinded to experimental conditions to insure unbiased quantification of the data. Densitometric quantification for Iba1-ir and GFAP-ir within the dorsal hippocampus or mPFC were performed using previously described methods (Williams and Milner 2011; Williams et al. 2011; Pierce et al. 2014). Sections were photographed with a Nikon Eclipse 80i microscope using a Micropublisher 5.0 digital camera (Q-imaging) and IPLab software (Scanalytics IPLab, RRID: SCR_002775). Average pixel density within the region of interest (ROI) was determined using ImageJ64 (ImageJ, RRID:SCR_003070) software. Using the Allen Brain atlas (https://portal.brain-map.org) as a guide, the dorsal hippocampus (between images 70 and 76) and mPFC (between images 35 and 39) regions were selected for analysis. ROIs within four subregions of the dorsal hippocampus were selected: (1) CA1: stratum radiatum (SR) and stratum lacunosum–moleculare (SLM); (2) CA2/3a: stratum oriens (SO), pyramidal cell layer (PCL), and near and distal SR; (3) CA3b: SO, PCL, stratum lucidum (SLu), and SR; and (4) DG: the supragranular blade (SG), the infragranular blade (IFG), and the central hilus (Cen). As described in our prior study (Rubin et al. 2020), two ROIs were selected from the mPFC using the corpus callosum as a guide: infralimbic (IL) and prelimbic (PL). Pixel density of a small region lacking labeling (i.e., corpus callosum or neuropil) was subtracted from ROI measurements to control for variations in illumination between images and to compensate for background labeling. Prior studies (Pierce et al. 2014) have shown a strong linear correlation between average pixel density and actual transmittance, demonstrating the accuracy of the technique. In ROIs that exhibited differences in Iba1 density, we determined whether these differences were due to changes in the density of processes or cell number (see Table 1). For this, the number of Iba1-labeled cells was counted in a 6.25-cm^2^ area in ROIs showing significant differences. As no differences in Iba1 density were seen in CA2/CA3a and the DG of males, Iba1 cell bodies were not counted in these regions in males.

Data are expressed as means ± SEM. Statistical analyses were conducted using JMP 12 Pro software (JMP, RRID: SCR_014242), and significance was set to an alpha <0.05. Differences in SBP between groups were compared by two-way analysis of variance (ANOVA) followed by Tukey post-hoc tests. Differences in glial markers within the hippocampus and mPFC were determined using a Student's *t*-test.

Images first were adjusted for contrast and sharpness in Adobe Photoshop 9.0 (Adobe Photoshop, RRID: SCR_014199). Next, images were imported into Microsoft PowerPoint 2010, where final adjustments to brightness, sharpness, and contrast were achieved. Adjustments were made to the entire image, none of which significantly altered the appearance of the initial raw image. Graphs were generated using Prism 8 software (Graphpad Prism, RRID: SCR_002798).

### Competing interest statement

The authors declare no competing interests.
